# Krill oil significantly decreases 2-arachidonoylglycerol plasma levels in obese subjects

**DOI:** 10.1186/1743-7075-8-7

**Published:** 2011-01-30

**Authors:** Sebastiano Banni, Gianfranca Carta, Elisabetta Murru, Lina Cordeddu, Elena Giordano, Anna Rita Sirigu, Kjetil Berge, Hogne Vik, Kevin C Maki, Vincenzo Di Marzo, Mikko Griinari

**Affiliations:** 1 Dipartimento Biologia Sperimentale, Università di Cagliari, Cittadella Universitaria, 09042 Monserrato (CA), Italy; 2 Nutrisearch s.r.l., Edificio 5 A1 Parco scientifico e tecnologico Polaris,09010 Pula, Italy; 3 Aker Biomarine ASA, Fjordallèen 16, NO-0115 Oslo, Norway; 4 Provident Clinical Research, 489 Taft Avenue, Glen Ellyn, Illinois 60137, USA; 5 Endocannabinoid Research Group, Institute of Biomolecular Chemistry, Consiglio Nazionale delle Ricerche, Via Campi Flegrei 34, Comprensorio Olivetti, 80078 Pozzuoli (NA), Italy; 6 Clanet Ltd., Kultarinnantie 1 b, Espoo, 02660, Finland

## Abstract

We have previously shown that krill oil (KO), more efficiently than fish oil, was able to downregulate the endocannabinoid system in different tissues of obese zucker rats.

We therefore aimed at investigating whether an intake of 2 g/d of either KO or menhaden oil (MO), which provides 309 mg/d of EPA/DHA 2:1 and 390 mg/d of EPA/DHA 1:1 respectively, or olive oil (OO) for four weeks, is able to modify plasma endocannabinoids in overweight and obese subjects.

The results confirmed data in the literature describing increased levels of endocannabinoids in overweight and obese with respect to normo-weight subjects. KO, but not MO or OO, was able to significantly decrease 2-arachidonoylglycerol (2-AG), although only in obese subjects. In addition, the decrease of 2-AG was correlated to the plasma n-6/n-3 phospholipid long chain polyunsaturated fatty acid (LCPUFA) ratio. These data show for the first time in humans that relatively low doses of LCPUFA n-3 as KO can significantly decrease plasma 2-AG levels in obese subjects in relation to decrease of plasma phospholipid n-6/n-3 LCPUFA ratio. This effect is not linked to changes of metabolic syndrome parameters but is most likely due to a decrease of 2-AG biosynthesis caused by the replacement of 2-AG ultimate precursor, arachidonic acid, with n-3 PUFAs, as previously described in obese Zucker rats.

## Introduction

The endocannabinoid system is deeply involved in the regulation of the homeostasis of body composition by regulating food intake and energy expenditure. An overactive endocannabinoid system was suggested to contribute to increased fat mass and to several features of metabolic syndrome [1]. In fact, an increase of anandamide (AEA) and 2-arachidonoylglycerol (2-AG) in overweight and obese subjects has been described [2-4].

A therapeutic approach aimed at re-establishing a physiological tone of the endocannabinoid system mainly relies on using antagonists of the one of their targets that is mostly responsible for their metabolic effects, i.e. the cannabinoid CB1 receptor [5]. However, it has been shown that the use of these antagonists in obese individuals is accompanied by psychiatric side effects such as increased incidence of depression and anxiety [5,6].

Endocannabinoids are ultimately derived from arachidonic acid incorporated in the *sn*-1 or *sn*-2 position of phospholipids, and their biosynthesis was shown to be affected by dietary fatty acids and in particular by EPA and DHA [7].

Recently [8], we have shown that in Zucker rats, an animal model of obesity, both krill oil (KO) and fish oil similarly increased EPA and DHA plasma levels, with KO being more effective than fish oil in improving some parameters of metabolic syndrome such as fatty liver and fatty heart. This might most probably be related to the stronger inhibitory effect of KO on endocannabinoid levels in these tissues and, particularly, in the visceral adipose tissue. In addition, we have recently reported that administration of 2 g/d of KO or menhaden oil (MO) for four weeks, significantly increased EPA and DHA levels in plasma in normal, overweight and obese subjects [9]. This KO dose provided 216 mg/d EPA and 90 mg/d DHA, while MO provided 212 mg/d EPA and 178 mg/d DHA. The International Society for the Study of Fatty Acids and Lipids (ISSFAL) reported as a recommendation [10] for cardiovascular health a minimum intake of combined EPA and DHA of 500 mg/day, based on several studies showing a significant reduction of cardiovascular risk with this dose or higher. In addition, the effect of n-3 long chain polyunsaturated fatty acids (LCPUFA) on metabolic syndrome parameters has been shown to be effective at much higher doses [11].

To our knowledge, the effect of dietary n-3 fatty acids on AEA and 2-AG concentrations in human plasma has never been investigated. This issue is not trivial, since it is well established that plasma 2-AG levels in obese individuals strongly correlate with several parameters of the metabolic syndrome, including visceral adipose tissue, high triglyceride levels, low HDL-cholesterol levels and indices of insulin resistance. Therefore, in this study we aimed at verifying whether or not four-week dietary intake of KO, FO or olive oil (OO), is able to modify endocannabinoid levels in the plasma of normo-weight, overweight and obese subjects.

## Materials and methods

### Study design

This was a 4-week, randomized, double-blind, controlled, parallel clinical trial conducted at 2 clinical research sites in the United States (Provident Clinical Research, Bloomington, IN, and Meridien Research, St. Petersburg, FL). The study included 3 visits: 2 screening/baseline visits (weeks -1 and 0) and 1 end-of-treatment visit (week 4). An independent institutional review board, Quorum Review, Inc. (Seattle, Wash), approved the protocol before initiation of the study, and written informed consent was obtained from all subjects before protocol-specific procedures were performed.

### Subjects

63 subjects generally healthy men and women, 35 to 64 years of age, with waist circumference of 102 cm or greater (men) or 88 cm or greater (women) were included (see Table 1 for demographic and anthropometric characteristics). Pregnant (or those planning to become pregnant during the study period) and lactating women were excluded. Volunteers who consumed fish more than 3 times in the month before screening were not eligible for enrollment and consumption of fish and seafood products was prohibited during the study. Individuals with a self-reported history of diabetes, inflammatory bowel disease, pancreatitis, and gallbladder or biliary disease in the 12 months before the screening visit were excluded from the study. In addition, those with a history of cancer (except for nonmelanoma skin cancer) in the 2 years before screening or any major trauma or surgical event within 3 months before screening were not enrolled. Volunteers were also excluded if they had serum triglycerides (TG) ≥500 mg/dL, total cholesterol (TC) ≥300 mg/dL, or uncontrolled hypertension (systolic blood pressure ≥160 mm Hg or diastolic blood pressure ≥100 mm Hg) at screening. The use of lipid-altering medications or supplements, non-study-related omega-3 fatty acid supplements (eg, flaxseed, fish, or algal oils) or omega-3 fatty acid-enriched or fortified foods, and anticoagulants was prohibited within 2 weeks of screening and throughout the study.

**Table 1:** Baseline demographic and anthropometric characteristics of subjects by treatment group.

	OO	MO	KO
	(n = 19)	(n = 23)	(n = 21)
Male, n	3	4	3
Female, n	16	19	18
Normoweight, n, male/female	1/3	1/3	1/6
Overweight, n, male/female	1/6	2/5	1/4
Obese, n, male/female	1/7	1/11	1/8
Age, year, mean ± SEM	47.4 ± 8.5	49.6 ± 8.7	49.4 ± 8.5
Body mass index, kg/m2, mean ± SEM	30.6 ± 1.3	31.6 ± 0.9	30.1 ± 1.0

Stratification of the subjects has been carried out by BMI values: normoweight BMI <25; overweight 25 < BMI <30; obese 30 < BMI <35.

There were no significant differences between groups for any variable.

### Study procedures

At baseline, eligible subjects were randomly assigned to 1 of 3 groups: 2 g/d of either KO (Superba krill oil, Aker BioMarine ASA, Oslo, Norway), MO (Omega-Pure, Houston, Tex), or olive oil (control). Subjects were instructed to consume four 500 mg capsules per day, preferably 2 capsules with each of 2 meals, for 4 weeks. Four capsules of the KO supplement provided 216 mg/d EPA and 90 mg/d DHA, and the MO supplement provided 212 mg/d EPA and 178 mg/d DHA. Stratification of the subjects has been carried out by BMI values: normoweight BMI <25; overweight 25 < BMI <30; obese 30 < BMI <35.

Further details of the study are described in [9].

### Lipid analyses

Total lipids were extracted from plasma using chloroform/methanol 2:1 (v/v) [12]. Separation of phospholipids (PL) from total lipids was performed as previously reported [13]. Aliquots were mildly saponified as previously described [14] in order to obtain free fatty acids for HPLC analysis. Separation of fatty acids was carried out with an Agilent 1100 HPLC system (Agilent, Palo Alto, Calif., USA) equipped with a diode array detector as previously reported [15]. *N*-arachidonoylethanolamine (anandamide, AEA) and 2-arachidonoylglycerol (2-AG) were measured as previously described [16].

### Statistical analyses

One way ANOVA with the Bonferroni test for post-hoc analyses was applied to evaluate statistical differences between groups. Whereas t-student test for paired samples was applied to detect significant differences between before and after treatment.

## Results

No changes in BMI, waist circumference, glycemia and insulinemia were detected after any of the treatments (data not shown).

**Figure 1:**
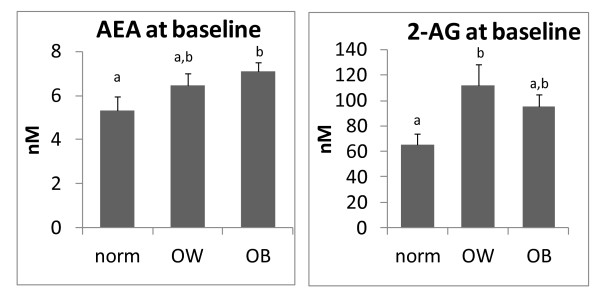
**Baseline Endocannabinoid plasma levels (nM), anandamide (AEA) left panel; 2-arachidonoylglycerol (2-AG) right panel, in normoweight subjects (norm) (n = 15), overweight (OW) subjects (n = 19) and obese subjects (OB) (n = 29)**. Error bars depict S.E.M. Different letters denote significant differences (p < 0.05).

**Figure 2:**
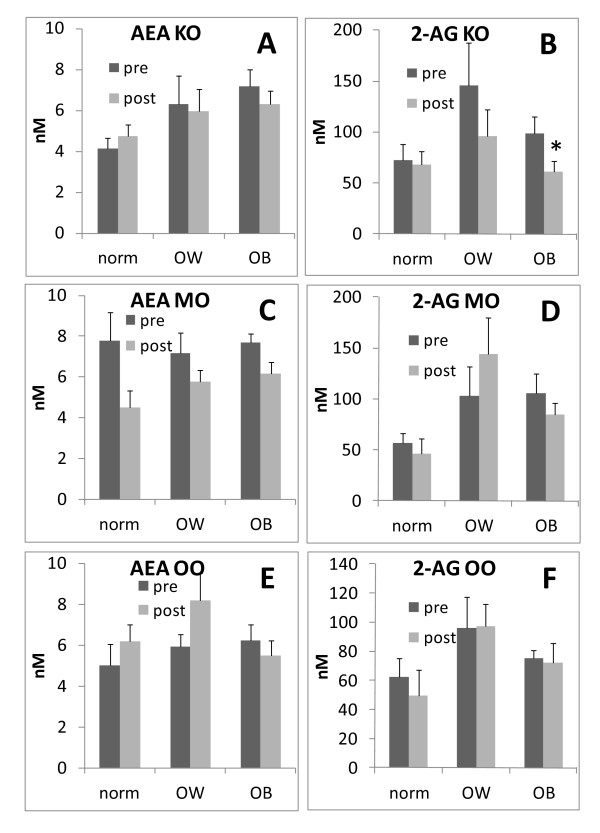
**Endocannabinoid plasma levels (nM) before (pre) and after (post) treatment with different oils**. norm = normoweight subjects; OW = overweight subjects; OB = obese subjects. **A **and **B **anandamide (AEA) and arachidonoylglycerol (2-AG) respectively with Krill oil (KO) treatment (n = 7, n = 5, n = 9 in norm, OW and OB respectively); **C **and **D **AEA and 2 AG respectively with menhaden oil (MO) treatment (n = 4, n = 7, n = 12 in norm, OW and OB respectively); **E **and **F **AEA and 2 AG respectively with olive oil (OO) treatment (n = 4, n = 7, n = 8 in norm, OW and OB respectively). Error bars depict S.E.M. * denotes statistical difference (p < 0.05).

**Figure 3:**
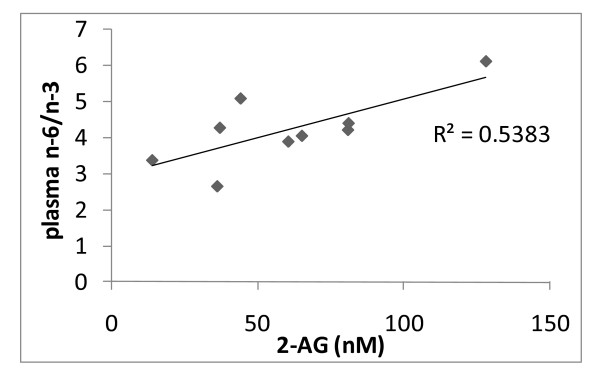
**Correlation between plasma phospholipid long chain PUFA n-6 (20:4+22:5+20:3+22:4)/long chain PUFA n-3 (20:5+22:6) ratio and 2-arachidonoylglycerol (2-AG). R**^**2 **^**= 0.55, p < 0.05**.

At baseline, plasma AEA levels were significantly higher in obese subjects, whereas plasma 2-AG levels were significantly higher only in overweight subjects (figure 1). Four week dietary intake of KO was able to significantly decrease 2-AG, but not AEA, only in the obese subjects, although a non-statistically significant trend towards a decrease was observed also in overweight subjects (figure 2). By contrast, MO or OO treatments did not modify endocannabinoid levels in either overweight or obese individuals (figure 2). A significant correlation between 2-AG levels and the plasma phospholipids n-6/n-3 LCPUFA ratio [(20:4n6+22:5n6+20:3n-6+22:4n-6)/(20:5n-3+22:6n-3)] was observed only in obese subjects whose diet was supplemented with KO (figure 3). No other correlation was found between endocannabinoids and single plasma phospholipid fatty acids, or in normo and overweight patients. In addition, due to the relatively low number of male subjects recruited, it was not possible to make any statistical analysis on gender differences in terms of treatment effects (data not shown).

## Discussion

In this pilot study we have confirmed data in the literature showing that overweight and obese subjects exhibit increased plasma levels of the endocannabinoids, AEA and 2-AG [2-4]. In our cohort of subjects, however, plasma 2-AG levels were increased significantly only in overweight individuals, whereas AEA levels were increased significantly only in obese subjects. This finding agrees with previous results suggesting that increased plasma AEA levels are associated with high BMI [17], whereas increased plasma 2-AG levels are associated with high visceral adipose tissue and not necessarily with high BMI [3,18,19]. It is possible that the cohort of obese subjects of the present study might have been characterized by a higher proportion of subcutaneous adipose tissue than in other cohorts previously investigated. As we did not acquire data on the adipose distribution in the obese subjects of a present study, this remains only a speculative hypothesis, to be specifically addressed in future studies. In addition, fat distribution in overweight premenopausal women may be different from that in postmenopausal women and in men for a given level of waist circumference. Thus, the small number of subjects in the present study and the heterogeneous nature of the sample (i.e., men as well as pre- and postmenopausal women) do not permit a meaningful assessment of the correlation of 2-AG levels with visceral fat distribution.

The novel finding of the present study is that KO, more efficiently than MO, was able to reduce endocannabinoid levels in the plasma despite the fact that the effects of the two dietary treatments on EPA and DHA plasma concentrations were comparable and even slightly lower in the KO group than in the MO group [9]. Comparable results were obtained in the visceral adipose tissue, liver and heart of obese Zucker rats [8]. However, in this previous study, 2-AG concentrations were decreased significantly by KO, and to a smaller extent by fish oil, only in the visceral adipose tissue. One possible explanation for the different effects of KO and fish oil might be, as previously suggested [8], the more efficient incorporation of n-3 LCPUFAs into visceral adipose tissue phospholipids, and subsequent decrease in arachidonic acid incorporation associated with KO supplementation, hence leading to impaired endocannabinoid biosynthesis.

Thus, it is tempting to suggest that plasma 2-AG mainly derives from this tissue, possibly because of its relatively high concentrations in this adipose depot. This hypothesis is in agreement with the strong correlations previously described between the amount of visceral adipose tissue and plasma 2-AG levels in overweight and obese subjects [18,19]. By contrast, in the subcutaneous adipose tissue of obese animals [20] and obese subjects with type 2 diabetes [2], 2-AG levels seem to be rather decreased, indicating that the 2-AG levels in the plasma cannot be predicted from those in the subcutaneous fat, and vice versa.

The positive correlation between 2-AG and the plasma phospholipid n-6/n-3 LCPUFA ratio, and not with the absolute plasma phospholipid concentrations of n-3 or n-6 LCPUFA, suggests that at least 2-AG levels are strongly influenced by fatty acid metabolism involving the balance between n-6 and n-3 LCPUFA. Interestingly, it has been demonstrated that the n-6/n-3 LCPUFA ratio, rather than absolute values of n-6 and n-3 PUFA, is correlated to cardiovascular disease [21], which is also directly associated with many of the metabolic disorders that positively correlate with plasma 2-AG levels [3,18,19]. Thus, it is tempting to hypothesize that KO ameliorates cardiovascular disorders in overweight and obese subjects, at least in part, by re-establishing a physiological endocannabinoid tone at CB1 receptors, via decrease of the n-6/n-3 phospholipid LCPUFA ratio and, hence, reduction of the ultimate biosynthetic precursors of 2-AG, the up-regulation of which is instead associated with visceral obesity, dyslipidemia, insulin resistance and atherogenic inflammation [5]. Since AEA is derived from AA esterified on the *sn*-1 position, and 2-AG from that esterified on the sn-2 position in the phospholipids, and since a reduction of the n-6/n-3 LCPUFA ratio would mostly affect the latter, this hypothesis, which is also based on the results from our previous study in Zucker rats, would also explain why KO only affected 2-AG and not AEA levels in the plasma. However, in the present study no significant differences in lipid metabolism, body weight or metabolic syndrome parameters were detected among the 3 groups of dietary treatments [9]. Therefore, the hypothesis that KO-induced reduction of plasma 2-AG levels may result in an amelioration of the metabolic dysfunctions associated with overweight and obesity will require further investigation. Even though a recent report [22] showed that plasma phospholipid n-3 PUFA was inversely associated with the metabolic syndrome, the lack of changes in metabolic syndrome parameters in the subjects that were administered with KO may suggest that four weeks of such treatment, and of the consequent KO-induced inhibition of 2-AG levels, is not sufficient to exert any beneficial metabolic effects. Indeed, even the direct antagonism of CB1 with rimonabant (20 mg/day) in obese subjects starts reducing body weight and ameliorating dyslipidemia and insulin resistance only after 2-3 months from the beginning of treatment [5]. Moreover, the lack of effect on triglyceride levels after treatment might be related to the fact that the participants in the study were normo-lipidemic. The lipid-lowering property of omega-3 fatty acids such as EPA and DHA is more pronounced in subjects with elevated triglycerides [23,24].

Future studies will have to investigate whether longer dietary interventions and higher dietary levels of KO, apart from still down-regulating the endocannabinoid system, also improve the metabolic syndrome, thus possibly representing an alternative to CB1 antagonists/inverse agonists for the treatment of this disorder.

## Competing interests

M.G., was a consultant for Aker Biomarine ASA, Oslo, Norway at the time of study. K.B. and H.V., are employed by Aker Biomarine ASA, Oslo, Norway. K.C.M. has received research funding and consulting fees from Aker Biomarine ASA, Oslo Norway. All other authors declare that they have no competing interests.

## Authors' contributions

SB, MG conceived of the study, participated in its design and supervision and drafted the manuscript with the contribution of all Authors; VD contributed to the interpretation of the data and supervised the analytical procedures; KB, HV evaluated the products used for the treatments and contributed to the study design; KCM supervised subject recruitment, qualification and treatment, blood sampling and clinical analyses; EG, EM, LC, GC, AS performed all analyses of plasma phospholipid fatty acid profile and plasma endocannabinoids, collected all data and made statistical analyses. All authors read, revised and approved the final manuscript.

## References

[B1] Matias I Petrosino S Racioppi A Capasso R Izzo AA Di Marzo V Dysregulation of peripheral endocannabinoid levels in hyperglycemia and obesity: Effect of high fat diets Mol Cell Endocrinol 2008 286 S66 78 10.1016/j.mce.2008.01.026 18343566

[B2] Annuzzi G Piscitelli F Di Marino L Patti L Giacco R Costabile G Bozzetto L Riccardi G Verde R Petrosino S Rivellese AA Di Marzo V Differential alterations of the concentrations of endocannabinoids and related lipids in the subcutaneous adipose tissue of obese diabetic patients Lipids Health Dis 2010 9 43 10.1186/1476-511X-9-43 20426869 PMC2868848

[B3] Di Marzo V Cote M Matias I Lemieux I Arsenault BJ Cartier A Piscitelli F Petrosino S Almeras N Despres JP Changes in plasma endocannabinoid levels in viscerally obese men following a 1 year lifestyle modification programme and waist circumference reduction: associations with changes in metabolic risk factors Diabetologia 2009 52 213 217 10.1007/s00125-008-1178-6 18972095

[B4] Sipe JC Scott TM Murray S Harismendy O Simon GM Cravatt BF Waalen J Biomarkers of endocannabinoid system activation in severe obesity PLoS One 2010 5 e8792 10.1371/journal.pone.0008792 20098695 PMC2808340

[B5] Di Marzo V Despres JP CB1 antagonists for obesity--what lessons have we learned from rimonabant? Nat Rev Endocrinol 2009 5 633 638 10.1038/nrendo.2009.197 19844251

[B6] Moreira FA Grieb M Lutz B Central side-effects of therapies based on CB1 cannabinoid receptor agonists and antagonists: focus on anxiety and depression Best Pract Res Clin Endocrinol Metab 2009 23 133 144 10.1016/j.beem.2008.09.003 19285266

[B7] Banni S Di Marzo V Effect of dietary fat on endocannabinoids and related mediators: consequences on energy homeostasis, inflammation and mood Mol Nutr Food Res 2010 54 82 92 10.1002/mnfr.200900516 20013888

[B8] Batetta B Griinari M Carta G Murru E Ligresti A Cordeddu L Giordano E Sanna F Bisogno T Uda S Collu M Bruheim I Di Marzo V Banni S Endocannabinoids may mediate the ability of (n-3) fatty acids to reduce ectopic fat and inflammatory mediators in obese Zucker rats J Nutr 2009 139 1495 1501 10.3945/jn.109.104844 19549757

[B9] Maki KC Reeves MS Farmer M Griinari M Berge K Vik H Hubacher R Rains TM Krill oil supplementation increases plasma concentrations of eicosapentaenoic and docosahexaenoic acids in overweight and obese men and women Nutr Res 2009 29 609 615 10.1016/j.nutres.2009.09.004 19854375

[B10] International Society for the Study of Fatty Acids and Lipids http://www.issfal.org.uk/images/stories/pdfs/PUFAIntakeReccomdFinalReport.pdf

[B11] Carpentier YA Portois L Malaisse WJ n-3 fatty acids and the metabolic syndrome Am J Clin Nutr 2006 83 1499S 1504S 16841860 10.1093/ajcn/83.6.1499S

[B12] Folch J Lees M Sloane Stanley GH A simple method for the isolation and purification of total lipides from animal tissues J Biol Chem 1957 226 497 509 13428781

[B13] Banni S Carta G Angioni E Murru E Scanu P Melis MP Bauman DE Fischer SM Ip C Distribution of conjugated linoleic acid and metabolites in different lipid fractions in the rat liver J Lipid Res 2001 42 1056 1061 11441132

[B14] Banni S Carta G Contini MS Angioni E Deiana M Dessi MA Melis MP Corongiu FP Characterization of Conjugated Diene Fatty Acids in Milk, Dairy Products, and Lamb Tissues J Nutr Biochem 1996 7 150 155 10.1016/0955-2863(95)00193-X

[B15] Melis MP Angioni E Carta G Murru E Scanu P Spada S Banni S Characterization of conjugated linoleic acid and its metabolites by RP-HPLC with diode array detector Eur J Lipid Sci Technol 2001 103 617 621 10.1002/1438-9312(200109)103:9<617::AID-EJLT6170>3.0.CO;2-C

[B16] Di Marzo V Goparaju SK Wang L Liu J Batkai S Jarai Z Fezza F Miura GI Palmiter RD Sugiura T Kunos G Leptin-regulated endocannabinoids are involved in maintaining food intake Nature 2001 410 822 825 10.1038/35071088 11298451

[B17] Engeli S Bohnke J Feldpausch M Gorzelniak K Janke J Batkai S Pacher P Harvey-White J Luft FC Sharma AM Jordan J Activation of the peripheral endocannabinoid system in human obesity Diabetes 2005 54 2838 2843 10.2337/diabetes.54.10.2838 16186383 PMC2228268

[B18] Bluher M Engeli S Kloting N Berndt J Fasshauer M Batkai S Pacher P Schon MR Jordan J Stumvoll M Dysregulation of the peripheral and adipose tissue endocannabinoid system in human abdominal obesity Diabetes 2006 55 3053 3060 10.2337/db06-0812 17065342 PMC2228260

[B19] Cote M Matias I Lemieux I Petrosino S Almeras N Despres JP Di Marzo V Circulating endocannabinoid levels, abdominal adiposity and related cardiometabolic risk factors in obese men Int J Obes (Lond) 2007 31 692 699 17224929 10.1038/sj.ijo.0803539

[B20] Izzo AA Piscitelli F Capasso R Aviello G Romano B Borrelli F Petrosino S Di Marzo V Peripheral endocannabinoid dysregulation in obesity: relation to intestinal motility and energy processing induced by food deprivation and re-feeding Br J Pharmacol 2009 158 451 461 10.1111/j.1476-5381.2009.00183.x 19371345 PMC2757684

[B21] Griffin BA How relevant is the ratio of dietary n-6 to n-3 polyunsaturated fatty acids to cardiovascular disease risk? Evidence from the OPTILIP study Curr Opin Lipidol 2008 19 57 62 10.1097/MOL.0b013e3282f2e2a8 18196988

[B22] Huang T Bhulaidok S Cai Z Xu T Xu F Wahlqvist ML Li D Plasma phospholipids n-3 polyunsaturated fatty acid is associated with metabolic syndrome Mol Nutr Food Res 54 1628 1635 10.1002/mnfr.201000025 20540149

[B23] Harris WS Fish oils and plasma lipid and lipoprotein metabolism in humans: a critical review J Lipid Res 1989 30 785 807 2677200

[B24] Harris WS n-3 fatty acids and serum lipoproteins: human studies Am J Clin Nutr 1997 65 1645S 1654S 9129504 10.1093/ajcn/65.5.1645S

